# Author Correction: Direct identification of Mott Hubbard band pattern beyond charge density wave superlattice in monolayer 1T-NbSe_2_

**DOI:** 10.1038/s41467-021-23418-z

**Published:** 2021-05-10

**Authors:** Liwei Liu, Han Yang, Yuting Huang, Xuan Song, Quanzhen Zhang, Zeping Huang, Yanhui Hou, Yaoyao Chen, Ziqiang Xu, Teng Zhang, Xu Wu, Jiatao Sun, Yuan Huang, Fawei Zheng, Xianbin Li, Yugui Yao, Hong-Jun Gao, Yeliang Wang

**Affiliations:** 1 School of Information and Electronics, MIIT Key Laboratory for Low-Dimensional Quantum Structure and Devices, Beijing Institute of Technology, Beijing, China; 2 State Key Laboratory of Integrated Optoelectronics, College of Electronic Science and Engineering, Jilin University, Changchun, China; 3 Institute of Physics, Chinese Academy of Sciences, Beijing, China; 4 Key Lab of Advanced Optoelectronic Quantum Architecture and Measurement (MOE) and School of Physics, Beijing Institute of Technology, Beijing, China

**Keywords:** Two-dimensional materials, Electronic properties and materials, Structure of solids and liquids

Correction to: *Nature Communications* 10.1038/s41467-021-22233-w, published online 30 March 2021.

The original version of this Article contained an error in Fig. 3c, in which the yellow dots were offset with respect to the background STM image. This has been corrected in both the PDF and HTML versions of the Article.

