# Supplementary material for: Direct identification of Mott Hubbard band pattern beyond charge density wave superlattice in monolayer 1T-NbSe2
Source: Nat Commun. 2021 Mar 30;12:1978. doi: 10.1038/s41467-021-22233-w (PMC8010100; doi:10.1038/s41467-021-22233-w)
Supplement: Supplementary file 1 — Supplementary Information [file 41467_2021_22233_MOESM1_ESM.pdf]

Supplementary Information

Direct identification of Mott Hubbard band pattern beyond charge density wave superlattice in monolayer 1T-$NbSe_2$

Liwei Liu[1*], Han Yang[1], Yuting Huang[2], Xuan Song[1], Quanzhen Zhang[1], Zeping Huang[1], Yanhui Hou[1], Yaoyao Chen[1], Ziqiang Xu[1], Teng Zhang[1], Xu Wu[1], Jiatao Sun[1], Yuan Huang[1,3], Fawei Zheng[4], Xianbin Li[2], Yugui Yao[4], Hong-Jun Gao[3], and Yeliang Wang[1]

[1]School of Information and Electronics, MIIT Key Laboratory for Low-Dimensional Quantum Structure and Devices, Beijing Institute of Technology, Beijing 100081, China.

[2]State Key Laboratory of Integrated Optoelectronics, College of Electronic Science and Engineering, Jilin University, Changchun 130012, China.

[3]Institute of Physics, Chinese Academy of Sciences, Beijing 100190, China.

[4]Key Lab of Advanced Optoelectronic Quantum Architecture and Measurement (MOE) and School of Physics, Beijing Institute of Technology, Beijing 100081, China.

*email: liwei.liu@bit.edu.cn

Table of Contents

Supplementary Fig. 1 Differentiated STM image for easy determination of ML 1T-$NbSe_2$ islands.
Supplementary Fig. 2 Line *dI/dV* spectra along one unit cell of CDW pattern, which contains top, hollow-1, and hollow-2 regions.
Supplementary Fig. 3 Typical point spectroscopy *dI/dV* at top, hollow-1, and hollow-2 regions along with bias-dependent *dI/dV* mapping.
Supplementary Fig. 4 First-principles calculated band structure of CDW phase of 1T-$NbSe_2$ under different conditions.
Supplementary Fig. 5 STM images shown in Fig. 3 without markers at each top site to provide clearer inspection.
Supplementary Fig. 6 STM images at -1.5 and +0.2 V with corresponding FFTs, showing CDW pattern and additional $\sqrt{3} \times \sqrt{3}$ R30° pattern.
Supplementary Fig. 7 Topographic image and the line plot showing the $\sqrt{3} \times \sqrt{3}$ R30° order.
Supplementary Fig. 8 STM images and corresponding FFT of the UHB orbital with different biases.
Supplementary Fig. 9 Isovalue plots of three maximally localized Wannier functions.

Supplementary Fig. 10 STM images of LHB (a) and UHB (b) at sample temperature of 77 K.

Supplementary Fig. 11 The observation of √3 × √3 R30° supermodulation from different $NbSe_2$ islands with rotation angles of 0° (a-d) and 11° (e-g) on BLG/SiC(0001).

Supplementary Fig. 12 2D triangular lattice antiferromagnets to the quantum spin liquid picture.

Supplementary Fig. 13 *dI/dV* on the bilayer graphene (BLG) on SiC(0001), showing a general V-shape feature with the Dirac point at -0.4 eV.

Supplementary Note 1 The discussion on the issue that the LHB is much higher than that of UHB in this work on ML 1T $NbSe_2$.

Supplementary Note 2 The discussion on the assignment of the state at +0.2 eV as the Mott upper Hubbard band rather than in-gap state originated from doping or local defect.

Supplementary Note 3 The discussion on using Top, Hollow-1, and Hollow-2 in Fig. 2.

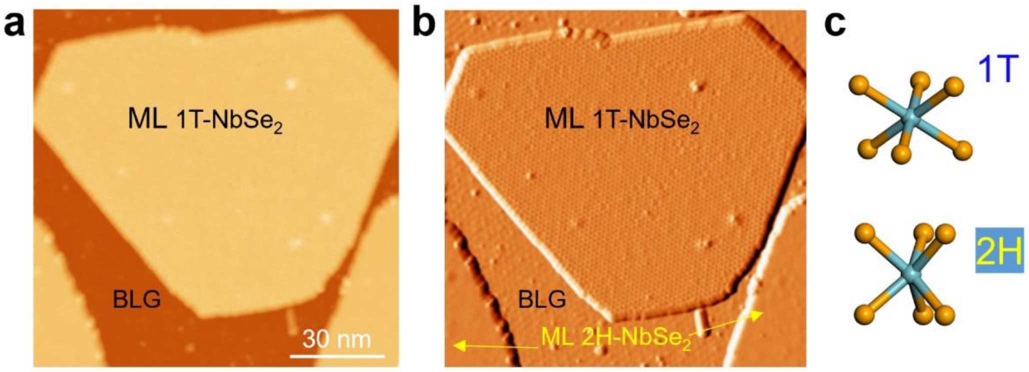

Supplementary Fig. 1 Differentiated STM image for determining ML 1T-$NbSe_2$ islands. a and b, raw and differentiated STM images of the same area (as shown in Fig. 1a in the main text). At sample temperature of 4.2 K, 1T and 2H $NbSe_2$ islands show different sizes in CDW patterns, i.e.,√13 × √13 R13.9° and 3 × 3, respectively. With differentiation, a significant fluctuation of the $NbSe_2$ surface arising from √13 × √13 R13.9° CDW pattern is conspicuous; this facilitates the determination of 1T $NbSe_2$ island. **c** atomic models for 1T and 2H phases.

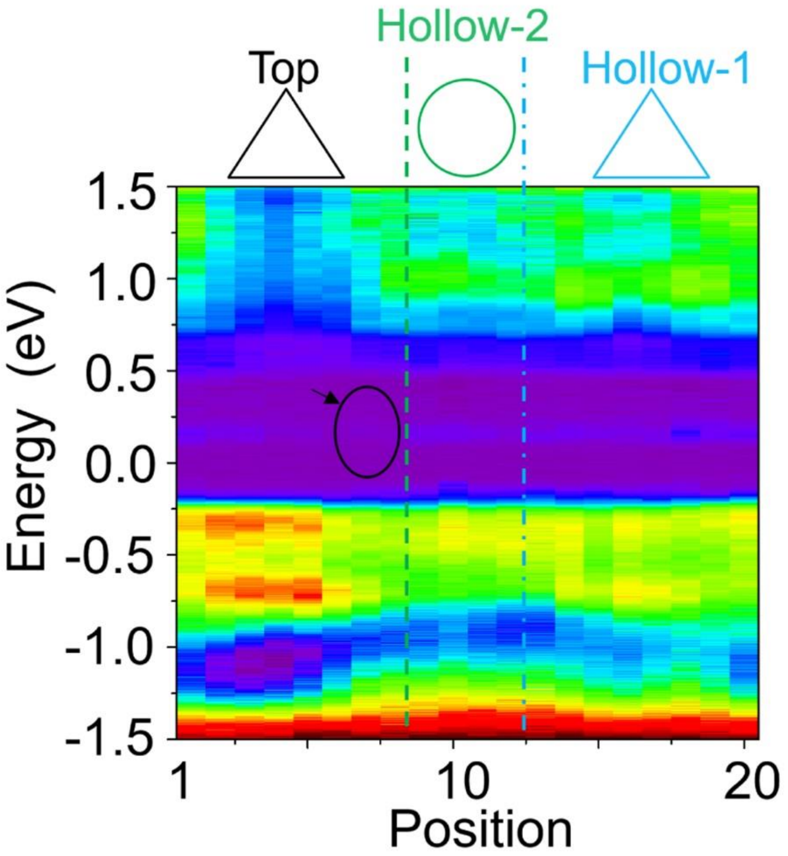

Supplementary Fig. 2 Line *dI/dV* spectra along one unit cell of CDW pattern, which contains top, hollow-1, and hollow-2 regions. At some part of top site (as indicated by the black ellipse and arrow), *dI/dV* intensity of +0.2 eV state was extremely weak.

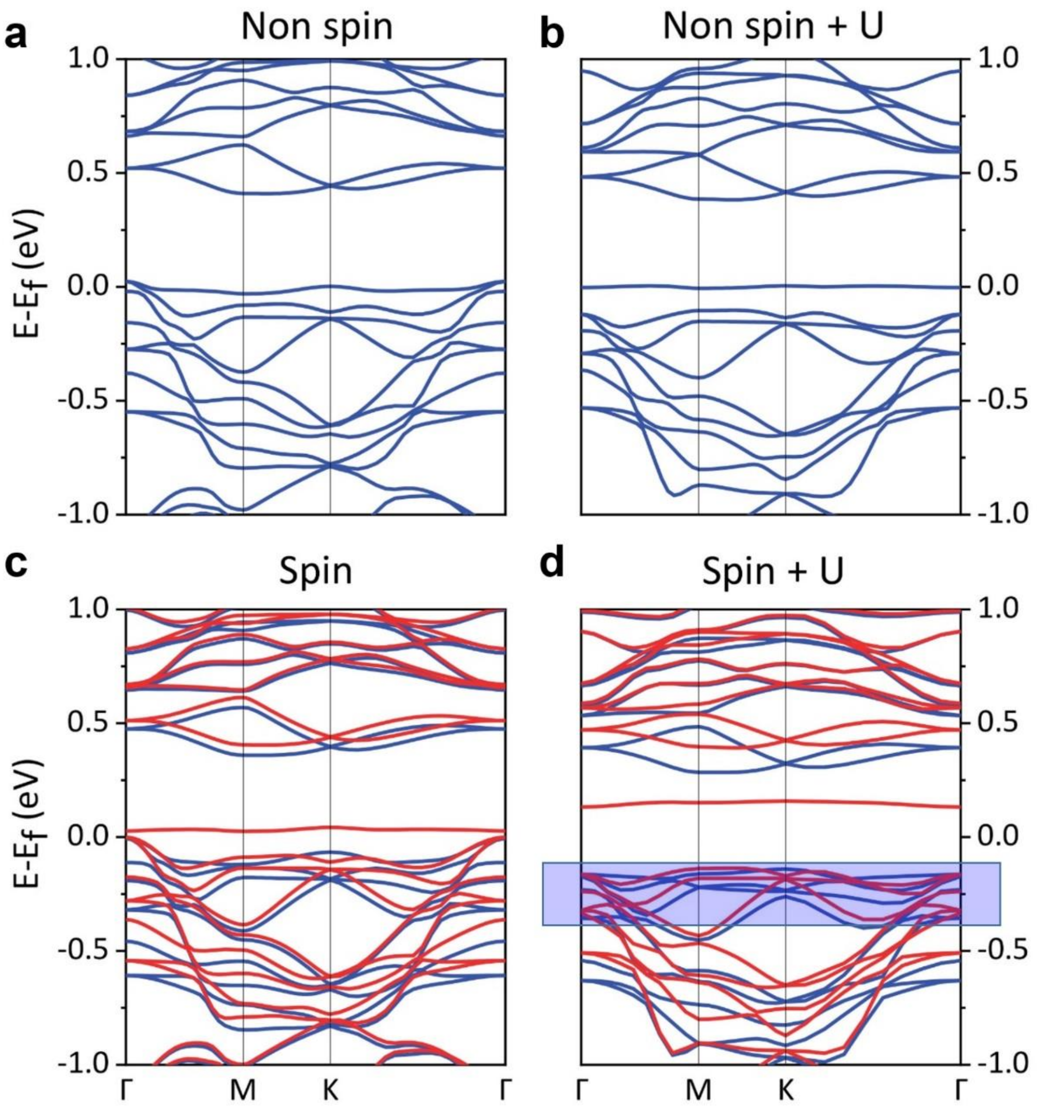

Supplementary Fig. 3 First-principles calculated band structure of charge density wave phase of 1T-$NbSe_2$ under different conditions. **a** Non-spin-polarized configuration; **b** Non-spin-polarized structure within GGA + U approximation. **c** Spin-polarized structure within GGA approximation. **d** Both spin polarization and GGA + U approximation considered; opening of Mott gap was due to the coupling of the Mott and magnetic effects. U was set to 2.8 eV.

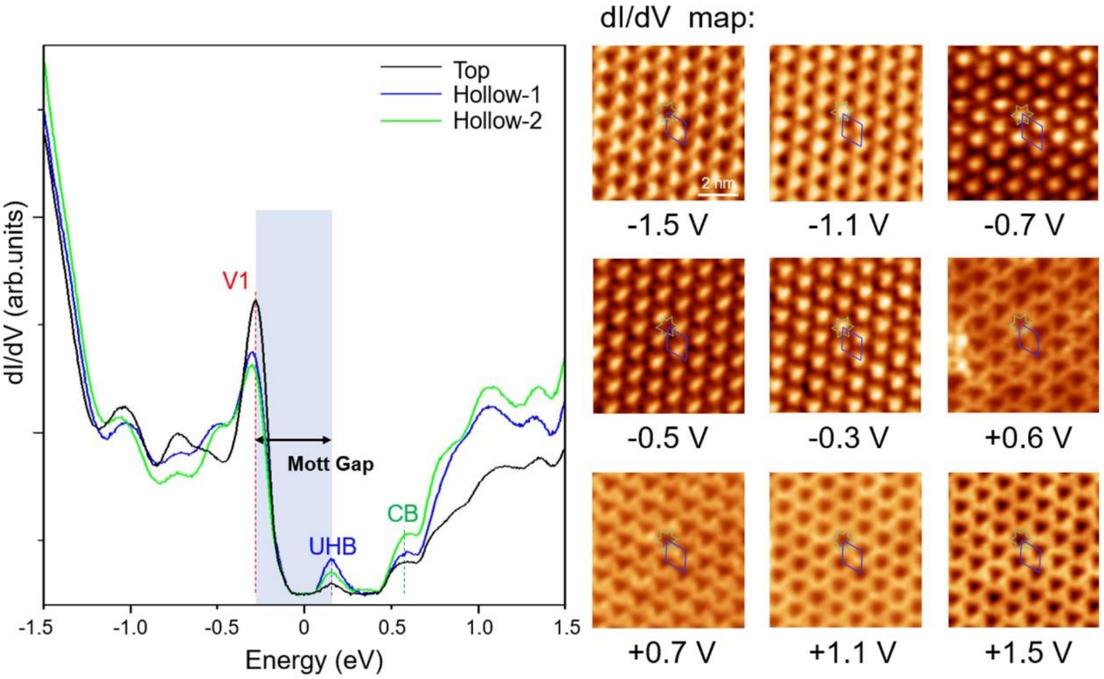

Supplementary Fig. 4 Typical point spectroscopy *dI/dV* spectra at Top, Hollow-1, and Hollow-2 regions along with bias-dependent *dI/dV* mapping. Mappings in the right panel indicate that top sites might exhibit bright or dark triangles at negative biases, although the top sites were always dark at positive biases.

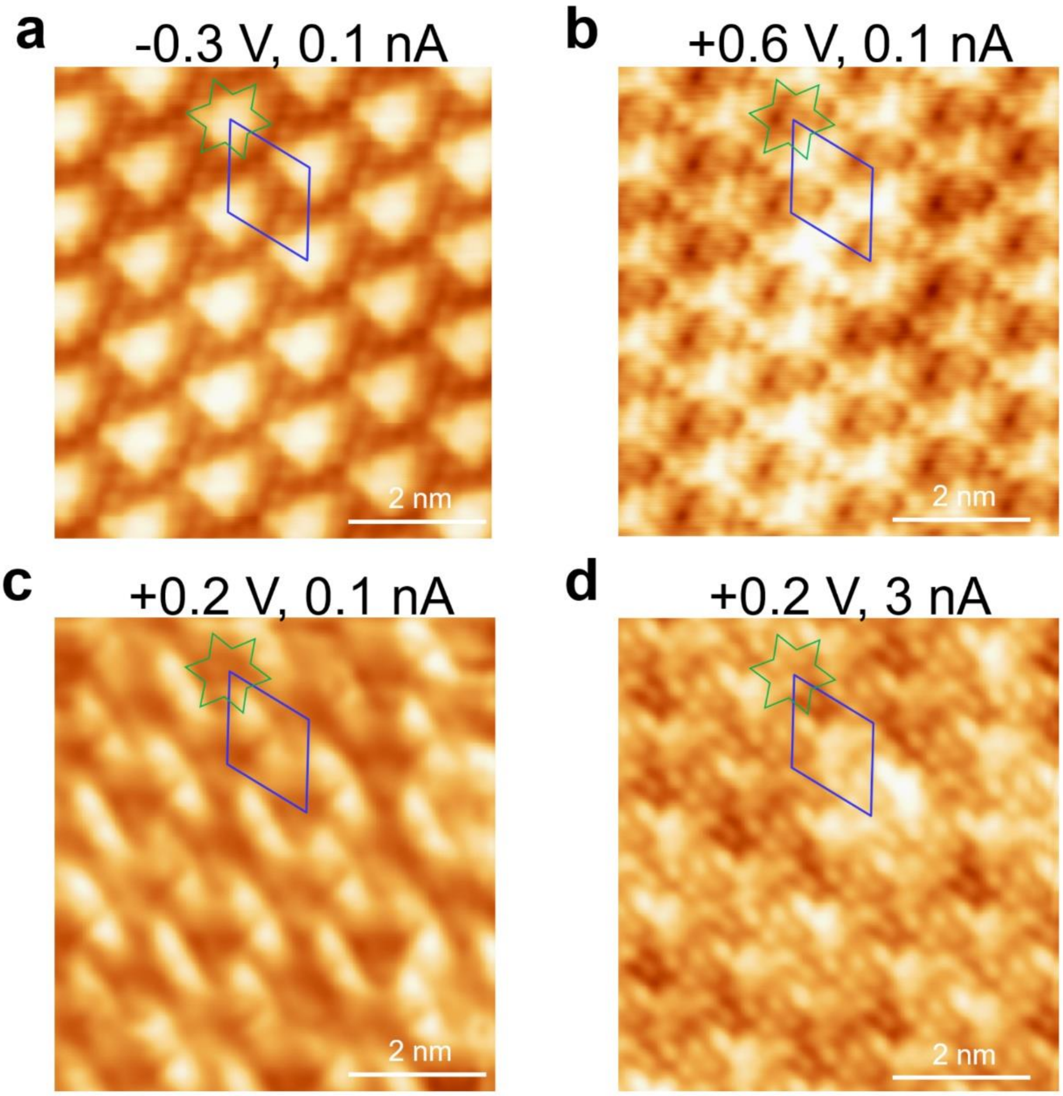

Supplementary Fig. 5 STM images shown in Fig. 3 without markers at top sites for clearer inspection.

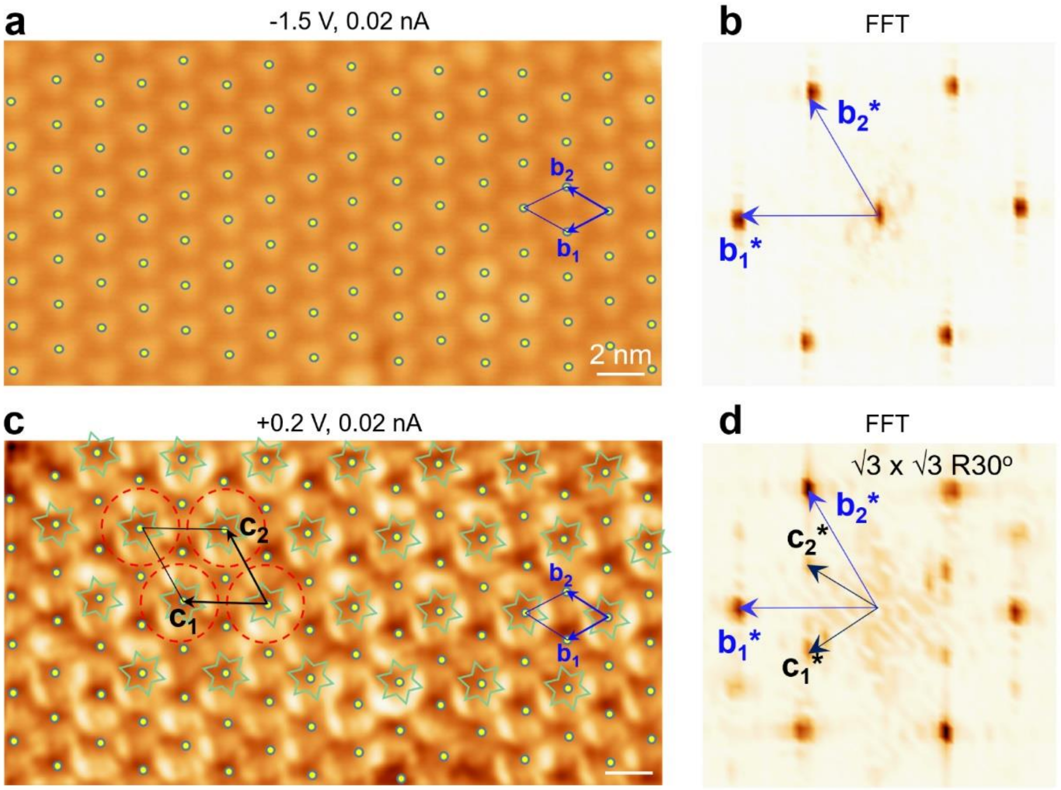

Supplementary Fig. 6 STM images at -1.5 and +0.2 V with corresponding FFTs, showing CDW pattern (with unit vectors b_1 and b_2) and additional √3 × √3 R30° pattern (with unit vectors c_1 and c_2). STM images in **a** and **c** captured at -1.5 and +0.2 V, the parts of which is shown in Fig. 3 in the main text. Reciprocal pattern and basis vectors are shown in **c** and **d**, respectively. An additional √3 ×√3 R30° of normal CDW pattern is shown at +0.2 V (UHB).

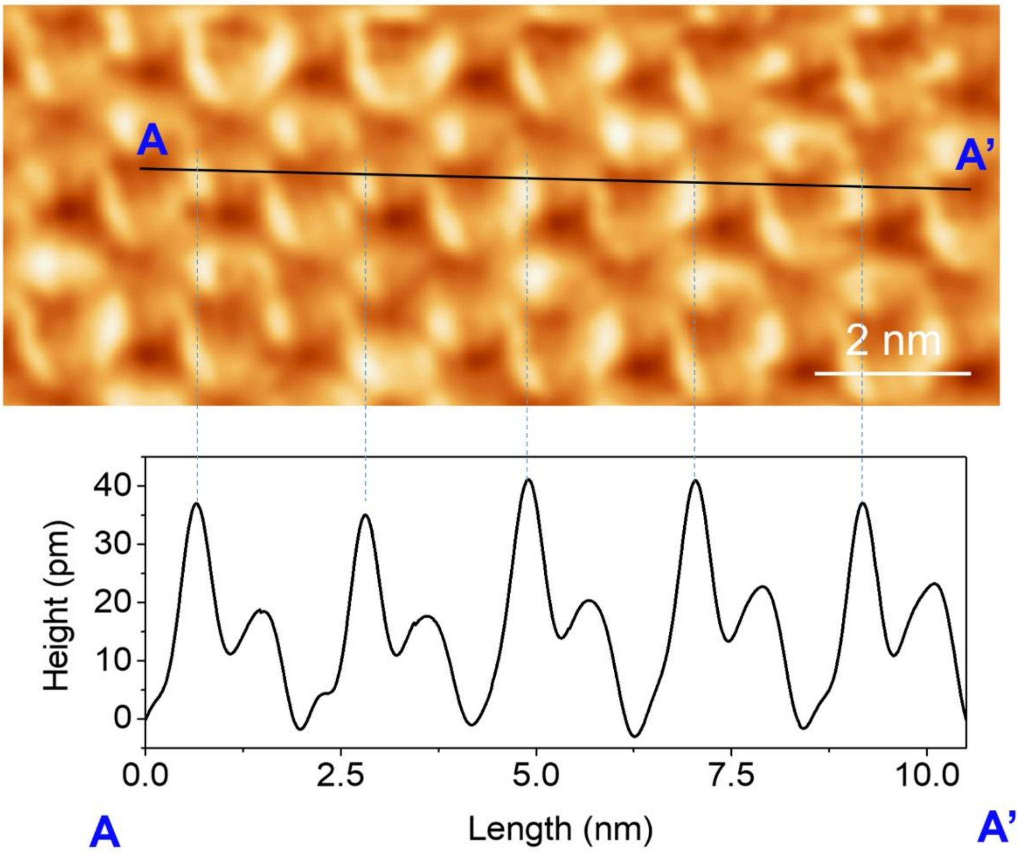

Supplementary Fig. 7 Topographic image and the line plot showing the √3 × √3 R30° order.

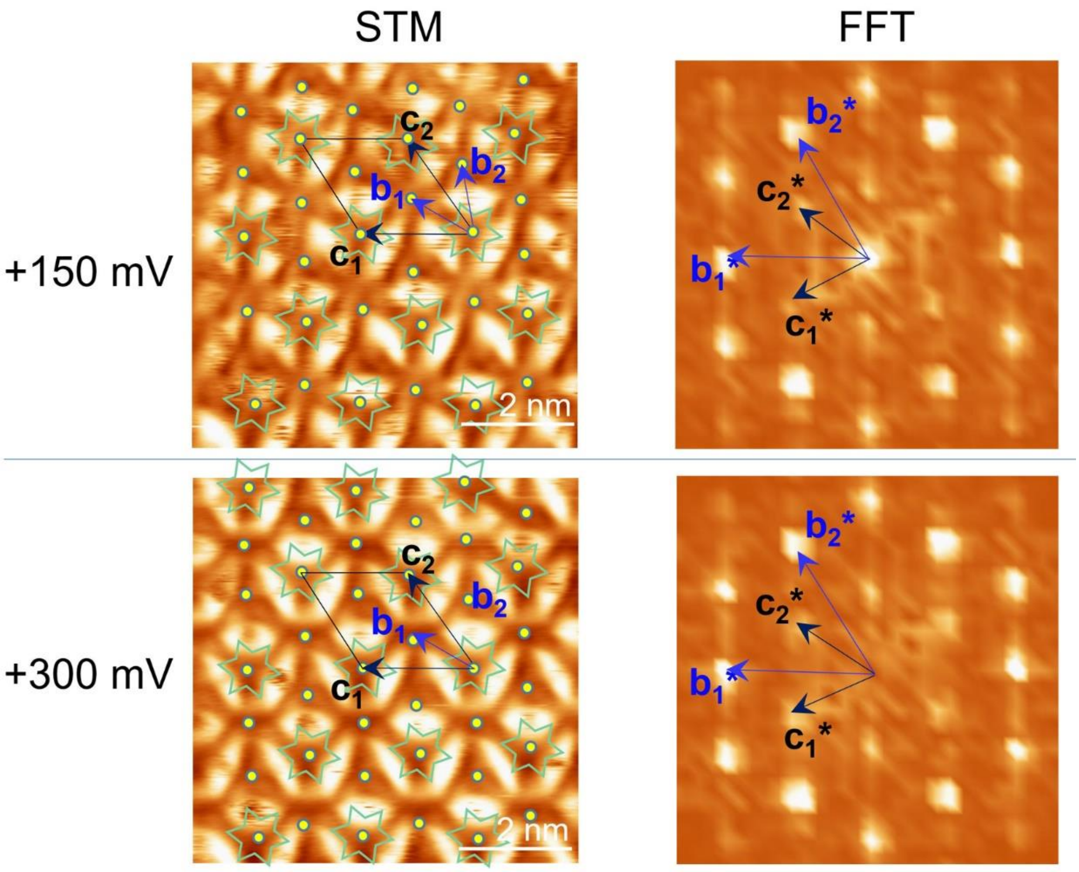

Supplementary Fig. 8 STM images and corresponding FFT of the UHB orbital with different biases. There is no evident change of root3 FFT peaks (c1* and c2*) at different bias voltages of 150 mV and 300 mV. The peaks (b1* and b2*) in FFT are originated from CDW superlattice.

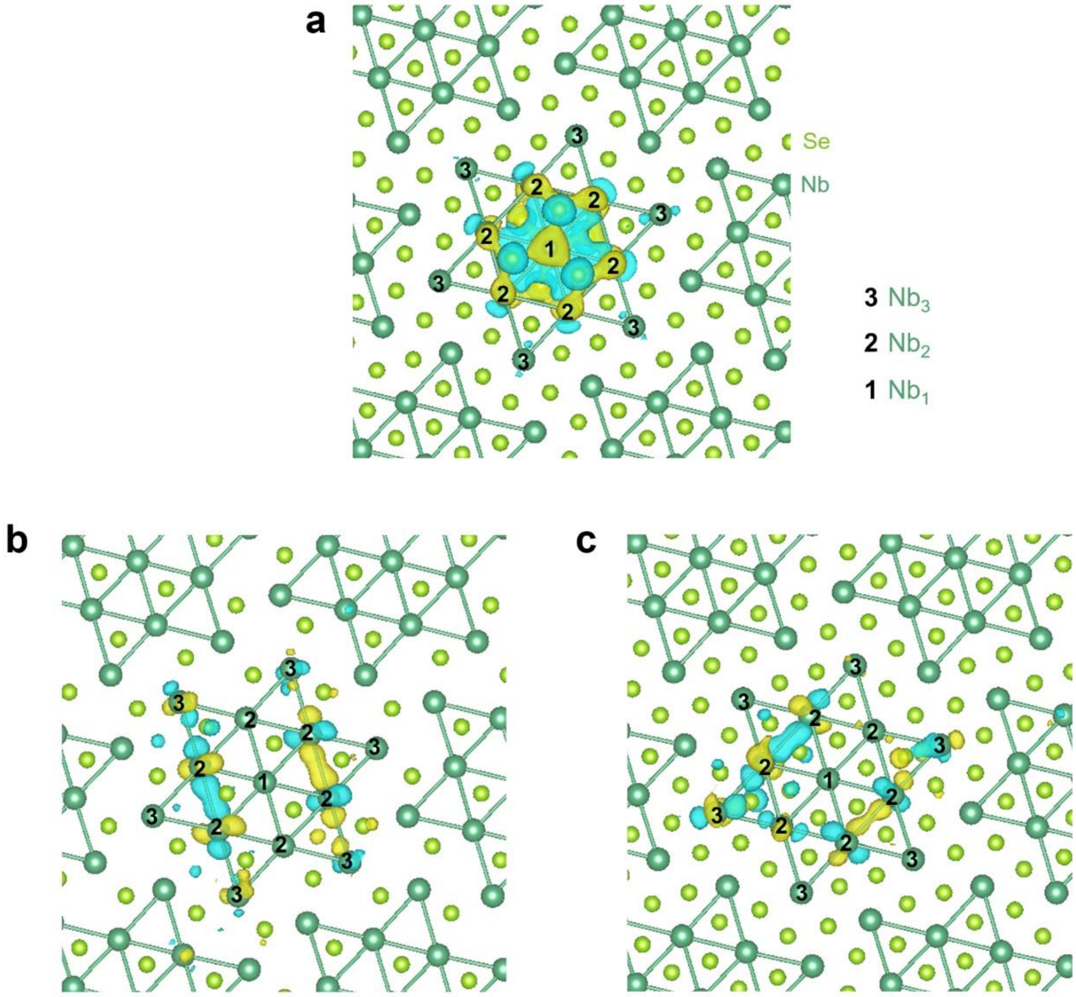

Supplementary Fig. 9 Isovalue plots of three maximally localized Wannier functions. **a** composed of the dz^2 orbital of the central Nb_1, the *d* orbitals of the surrounding Nb_2 and Nb_3, and the *p* orbitals of Se atoms. **b**, **c** composed of the *d* orbitals of the surrounding Nb_2 and Nb_3.

The Wannier functions for minimal three-band tight-binding model describing the electronic structures around Fermi level are obtained by using Wannier90 code[1], they are mainly contributed by the *d*-orbitals at the center and surrounding Nb atoms. In one single SOD, 13 Nb atoms were categorized as the central atom (Nb_1), six at the nearest sites (Nb_2), and the other six at the next nearest sites (Nb_3).

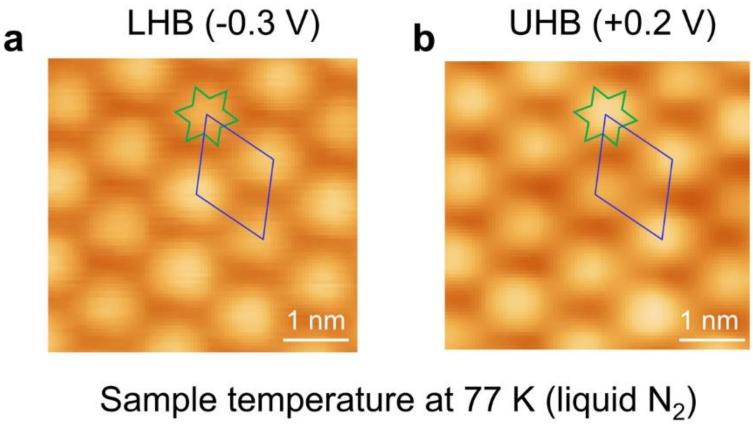

Supplementary Fig. 10 STM images of LHB (a) and UHB (b) at sample temperature of 77 K. Scanning parameters: **a**, bias voltage V_B = -0.3 V and V_B = 0.2 V for **a** and **b**, respectively; tunneling current I_t = 20 pA. The UHB orbital texture in (b) has the same spatial distribution as the Star of David CDW pattern (a), that is, both images show protrusions at the center of Star of David, different from the reversal spatial distribution while at sample temperature of 4.2 K.

We have further investigated the temperature dependence of the LHB and UHB orbital features. At a sample temperature of 4.2 K, while the LHB orbital texture follows the Star of David CDW pattern (top sites), the UHB shows a reversal spatial distribution. At 77 K, both LHB and UHB show normal spatial distribution (orbital texture is the same as the Star of David CDW top sites). We find this phenomenon keeps up to room temperature. Thus, the unexpected reversed spatial relationship of UHB is a low-temperature phenomenon, and probably relate to a 120° antiferromagnet to quantum spin liquid transition from 4.2 K to 77 K. The normal spatial distribution at 77 K further confirm the Mottness of the 1T-$NbSe_2$ (see Ref. 39, Kamil, E., *et al*, Electronic structure of single layer 1T-$NbSe_2$: interplay of lattice distortions, non-local exchange, and Mott-Hubbard correlations. *J. Phys.: Condens. Matter* 30, 325601 (2018) and Ref. 21 Qiao, S., *et al*. Mottness Collapse in 1T-TaS2-xSex Transition- Metal Dichalcogenide: An Interplay between Localized and Itinerant Orbitals. Phys. Rev. X, 7, 041054 (2017)).

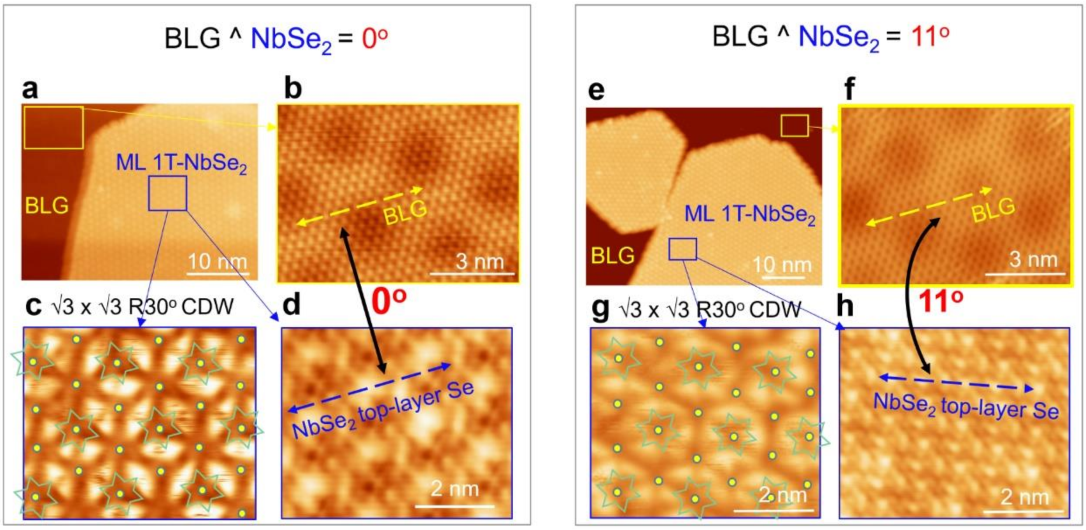

Supplementary Fig. 11 The observation of √3 × √3 R30° supermodulation from different $NbSe_2$ islands with rotation angles of 0° (a-d) and 11° (e-g) on BLG/SiC(0001). a, **e** $NbSe_2$ islands with different rotation angles with respect to BLG. **b**, **f** The atomic lattice of BLG next to the $NbSe_2$ islands. **d**, **h** The atomic lattice of $NbSe_2$. While the directions of the atomic lattice of BLG are the same, the directions of the atomic lattice of ML 1T-$NbSe_2$ are different: (0° and 11° with respect to the BLG lattice). **c**, **g** Both of the two islands reveal a √3 × √3 R30° pattern. Scanning parameters: **a**, **e** -1.5 V, 20 pA. **b**, **f** V_B = -1.5 V, I_t = 60 pA. **d**, **h** 200 mV, 6 nA. **c**, **g** 200 mV, 20 pA. Both of the two islands with inequivalent rotation angles with the BLG substrate reveal a √3 × √3 R30° pattern (c and g), thus we can exclude the commensurate moire structure as the reason for the √3 × √3 R30° supermodulation.

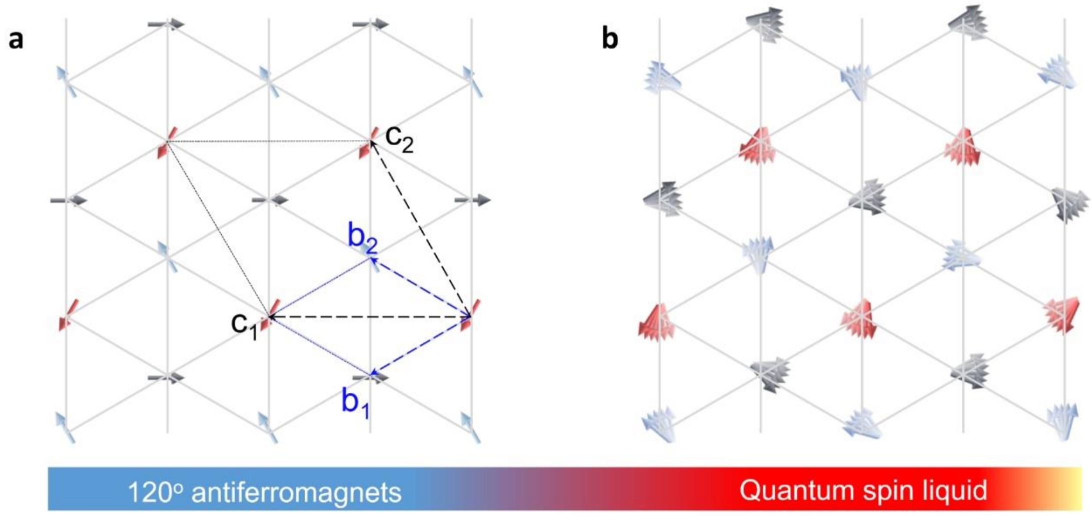

Supplementary Fig. 12 Heisenberg spin-1/2 antiferromagnets to the quantum spin liquid picture in 2D triangular lattice. The √3 × √3 superstructure (with unit vectors of c_1 and c_2) is local, leaving other areas as the quantum spin liquid due to the activation by next-nearest neighboring interaction.

As our GGA+U calculations show that the UHB is spin-polarized, each start-of-David (SOD) unit contains one spin at the center Nb atom (numbered as "1" in Fig. 1f in the main text), and the SOD cluster formed a periodic triangular lattice (see Huse, D. A. *et al.*, Simple Variational Wave-Functions for Two-Dimensional Heisenberg Spin-1/2 Antiferromagnets. Phys. Rev. Lett. 1988, 60, 2531-2534). Hence, the √3 × √3 superstructure in ML-1T $NbSe_2$ can be understood in the **Heisenberg spin-1/2 antiferromagnets picture in 2D triangular lattice**. In this picture, the ground state has three sublattices, and the spins on each sublattice are at an angle of 120° to the nearest neighbors (labeled by black, red, and blue arrows in Supplementary Fig. 8a), but are the same as its next nearest neighbors at distance √3 times of the nearest-neighbor spacings. The repetition of the SOD clusters with the same spin gives rise to the √3 × √3 superstructure, similar to the electron scattering and interference in the graphene lattice (Rutter, G. M., *et al.*, Scattering and interference in epitaxial graphene. Science 2007, 317, 219-222). Due to the activation by next-nearest neighboring interaction, the √3 × √3 superstructure is local, leaving other areas as the quantum spin liquid.

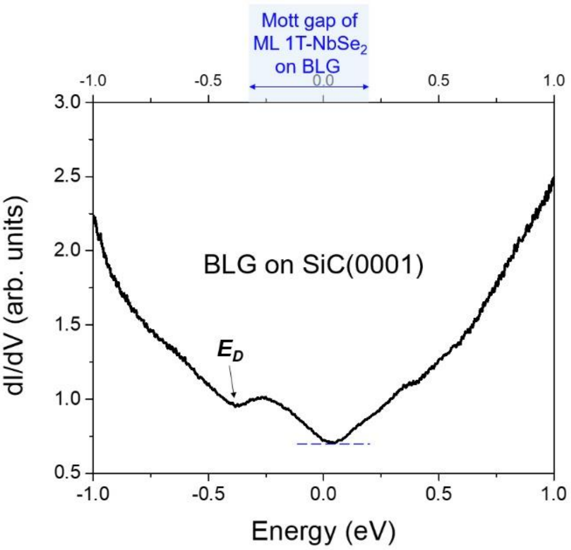

Supplementary Fig. 13 ***dI/dV*** **on the bilayer graphene (BLG) on SiC(0001), showing a general V-shape feature with the Dirac point at -0.4 eV.** Spectroscopy parameters: -1.5 V, 1 nA before turning off feedback loop.

For the bilayer graphene (BLG) substrate, the electronic states do not vanish to zero (zero conductance denoted by the dashed line) within the Mott gap of ML 1T-$NbSe_2$ (-0.3 eV~+0.2 eV). The imaging of the CDW pattern in the Mott gap is probably due to weak charge transfer between the BLG substrate and the $NbSe_2$ monolayer. As the majority of studies of atomic layers of transition-metal-dichalcogenides (TMDs) are grown on BLG/SiC(0001) surface, our results will be of general interest to the 2D material community.

Supplementary Notes

Supplementary Note 1 The discussion on the issue that the LHB is much higher than that of UHB in this work on ML 1T $NbSe_2$.

In our case, the LHB in *dI/dV* spectra is much higher than that of UHB. It is probably due to that the LHB is mixed with VB (named as LHB+VB) by the hybridization of the spin-up state with other orbitals.

Supplementary Note 2 The discussion on the assignment of the state at +0.2 eV as the Mott upper Hubbard band rather than in-gap state originated from doping or local defect.

An in-gap state can arise from an external effect like doping or local defect. However, based on the homogeneous spatial appearance in the +0.2 eV state (Figs. 2c and 2d) and the defect-free STM topography along the AA’ line, we can safely exclude the doping and defect states as the origin. Therefore, we attributed the +0.2 eV state as the UHB, also supported by STM imaging

obtained at sample temperature of 77 K (Supplementary Fig. 10), in line with a Mottness picture (Ref. 21).

Supplementary Note 3 The discussion on using Top, Hollow-1, and Hollow-2 in Fig. 2.

We use Nb1, Nb2 and Nb3 to distinguish three sites of Nb, and to emphasize the Nb1 at the center of the SOD plays an important role in the CDW formation and the isolated dz^2 orbital texture. This is mainly from the theoretical point of view, which considers the middle plane of the TMDs.

On the other hand, we use top, hollow-1, and hollow-2 in Fig. 2 to describe three kinds of areas, which can be distinguished well in STM images, and illustrate local contrast well in images.

References:

1. wannier90: A tool for obtaining maximally-localised Wannier functions, AA Mostofi, JR Yates, YS Lee, I Souza, D Vanderbilt, N Marzari, *Comput. Phys. Commun.* 178, 685 (2008)
